# GH3 phylogenetic subgroups define divergent routes of auxin inactivation via aspartate and glutamine conjugation

**DOI:** 10.1093/plphys/kiag412

**Published:** 2026-06-19

**Authors:** Lenka Helusová, David Ušák, Zuzana Havránková, Petre I Dobrev, Barbora Svobodová, Julie Talpová, Federica Brunoni, Tomáš Moravec, Karel Müller, Jan Petrášek

**Affiliations:** Institute of Experimental Botany of the Czech Academy of Sciences, Prague 6, Czech Republic; Department of Experimental Plant Biology, Faculty of Science, Charles University, Prague 2, Czech Republic; Institute of Experimental Botany of the Czech Academy of Sciences, Prague 6, Czech Republic; Department of Experimental Plant Biology, Faculty of Science, Charles University, Prague 2, Czech Republic; Institute of Experimental Botany of the Czech Academy of Sciences, Prague 6, Czech Republic; Institute of Experimental Botany of the Czech Academy of Sciences, Prague 6, Czech Republic; Institute of Experimental Botany of the Czech Academy of Sciences, Prague 6, Czech Republic; Institute of Experimental Botany of the Czech Academy of Sciences, Prague 6, Czech Republic; Laboratory of Growth Regulators, Faculty of Science, Palacký University & Institute of Experimental Botany of the Czech Academy of Sciences, Olomouc, Czech Republic; Institute of Experimental Botany of the Czech Academy of Sciences, Prague 6, Czech Republic; Institute of Experimental Botany of the Czech Academy of Sciences, Prague 6, Czech Republic; Institute of Experimental Botany of the Czech Academy of Sciences, Prague 6, Czech Republic

## Abstract

Auxin conjugation represents a key metabolic mechanism in regulating auxin activity within plant cells. GRETCHEN HAGEN3 (GH3) enzymes conjugate the major naturally occurring auxin indole-3-acetic acid (IAA) with amino acids, thereby contributing to the maintenance of auxin homeostasis. Although the transcription of *GH3* genes is auxin regulated, the complexity of this regulation is still not fully understood. Therefore, in this study, we employed β-estradiol-inducible and CRISPR/Cas9-mediated knock-out transgenic tobacco (*Nicotiana tabacum*) cell lines with modified expression of representative genes from 2 *GH3* subgroups with contrasting responses to auxin, *NtGH3.1a*, and *NtGH3.6e*. Using IAA metabolite profiling and bacterial enzyme assays, we show that NtGH3.1a preferentially catalyzes the formation of indole-3-acetyl-aspartate (IAA-Asp), while NtGH3.6e facilitates the production of the less-characterized conjugates indole-3-acetyl-glutamine (IAA-Gln) and 2-oxindole-3-acetyl-glutamine (oxIAA-Gln). We further validated these results by testing the *Arabidopsis thaliana* homologs of both GH3 subgroups, showing that AtGH3.1 favors aspartate, while both AtGH3.5 and AtGH3.6 preferentially utilize glutamine. Finally, subcellular localization analyses using green fluorescent protein (GFP)-tagged NtGH3.1aT and NtGH3.6eT expressed under inducible promoters demonstrated that both enzymes localized to the nucleus and cytoplasm, independently of the presence of auxin. Moreover, we provide evidence of GH3 localization under native promoters, confirming their presence in both compartments. Collectively, our results suggest the evolutionary conservation of amino acid type–preferential IAA conjugation and underscore the functional divergence of GH3 isoforms in IAA metabolism.

## Teaser

GRETCHEN HAGEN3 enzymes conjugate auxin to different amino acids according to their phylogenetic subgroups.

## Introduction

Indole-3-acetic acid (IAA), a major auxin, is one of the most important signal molecules in plants; therefore, IAA levels require precise native regulation, mainly through metabolism and transport. Metabolism of IAA comprises biosynthesis and inactivation processes, such as conjugation and oxidation (Hayashi et al. 2021; Vanneste et al. 2025). To this date, evidence has been found for IAA conjugation with amino acids (AAs), sugars, methyl esters, or peptides. Conjugation with sugars is mediated by UDP-GLYCOSYL TRANSFERASEs (UGTs), representing the main IAA inactivation pathway in *Arabidopsis thaliana* and *Zea mays* (Ostrowski et al. 2020; Mateo-Bonmatí et al. 2021). In relation to auxin, the most studied UGTs are AtUGT84B1 and AtUGT74D1 (Jin et al. 2013; Tanaka et al. 2014; Aoi et al. 2020; Mateo-Bonmatí et al. 2021), which can glycosylate both IAA to indole-3-acetyl-1-glucosyl ester (IAA-GE) and 2-oxindole-3-acetic acid (oxIAA) to 2-oxindole-3-acetyl-1-glucosyl ester (oxIAA-GE), and ZmIAGlc synthase (Ostrowski et al. 2020).

The *GRETCHEN HAGEN3 (GH3)* gene family codes for acyl acid amido synthetases, which catalyze the amino acid conjugation of IAA. Originally, the *GH3* genes were identified as rapid auxin-response genes, similarly to the *SAUR* and *AUX/IAA* gene families (Hagen et al. 1984; Hagen and Guilfoyle 2002). In addition to IAA, GH3s can conjugate amino acids to other plant hormones. For 2 decades, GH3s were divided into 3 groups according to their substrate phytohormone specificity and sequence homology: group I conjugating AAs to jasmonic acid (JA), group II to IAA, and group III to benzoates (Staswick et al. 2005; Holland and Jez 2024). An increasing number of studies have demonstrated that individual GH3s exhibit promiscuity to multiple phytohormones (Chen et al. 2010; Gutierrez et al. 2012; Westfall et al. 2016; Sherp et al. 2018; Brunoni et al. 2023; Hladík et al. 2025; Široká et al. 2025).

Inactivation of IAA through conjugation is reversible, and for each IAA conjugate, different cleaving enzymes were characterized. Hydrolases of sugar-linked IAA conjugates were identified in maize (Jakubowska and Kowalczyk 2005) and in rice (Ishimaru et al. 2013). However, to date no *Arabidopsis thaliana* homologs have been described. Hydrolysis of IAA conjugates with AAs (IAA-AA) is mediated by the IAA-LEUCINE-RESISTANT1 (ILR1) and ILR1-like (ILL) family of amidohydrolases, which can release active IAA (Bartel and Fink 1995; LeClere et al. 2002; Hayashi et al. 2021), JA (Widemann et al. 2013) and *cis*-(+)-12-oxo-phytodienoic acid (OPDA; Široká et al. 2025) from corresponding amino acid conjugates. Finally, oxidation of IAA has been recently described as closely related to AA conjugation, as IAA-AA conjugates are oxidized to 2-oxindole-3-acetyl-amino acid conjugates (oxIAA-AA). This process is mediated by DIOXYGENASE FOR AUXIN OXIDATION (DAO1), and in comparison to GH3-controlled conjugation, this reaction is irreversible (Hayashi et al. 2021; Müller et al. 2021).

The tissue-specific localization of GH3 proteins *in planta* was shown based on studies utilizing native *GH3* promoter–driven GUS or fluorescent proteins (Hagen et al. 1991; Yi et al. 1999; Takase et al. 2004; Di Mambro et al. 2017; Pierdonati et al. 2019; Wang et al. 2023). Notably, the widely used DR5 auxin-responsive reporter was originally derived from *GH3* promoter elements, which may explain some of the tissue-specific patterns observed in *GH3* expression studies (Ulmasov et al. 1997). The subcellular localization of GH3s in translational fusion was reported in the cytoplasm for PpGH3.1-GFP from *Physcomitrium patens* protoplasts (Ludwig-Müller et al. 2009), and in the cytoplasm and nucleus for GH3s in Arabidopsis or rice protoplasts and roots, AtGH3.3, AtGH3.5, and OsGH3.5 (Zhang et al. 2015; Liu et al. 2025; Široká et al. 2025). In all of these reports, GH3s were expressed under artificial CaMV 35S or UBQ10 promoters. However, data on the localization of GH3 under natural promoters are still missing.

The *GH3* genes were identified as early auxin-responsive genes (Hagen et al. 1984; Hagen and Guilfoyle 2002). In our previous study, we revealed that some *GH3* genes differ in their response to auxin (Müller et al. 2021). Here, using phylogenetic analysis, we describe in detail the diversity within the tobacco (*Nicotiana tabacum*) *GH3* gene family. We selected representatives of 2 main auxin-related GH3 subgroups and compared them for their substrate preference and intracellular distribution using cells of tobacco BY-2 (*Nicotiana tabacum* L. cv Bright Yellow) as a model system. The GH3.1-like subgroup has a higher affinity to aspartate (Asp), while the GH3.6*-*like subgroup seems to be involved in producing indole-3-acetyl-glutamine (IAA-Gln), which is subsequently oxidized to 2-oxindole-3-acetylglutamine (oxIAA-Gln), recently reported in a non–peer-reviewed study as an IAA metabolite (Dobrev et al. 2023). Selected candidates from both subgroups, NtGH3.1aT and NtGH3.6eT, were localized in both the cytoplasm and nucleus, and their localization was not influenced by the presence of auxin. The findings of this study corroborate the massive complexity involved in regulating free IAA activity through metabolic pathways in cells.

## Results

### The tobacco group II GH3s form 3 subgroups

**Figure 1:**
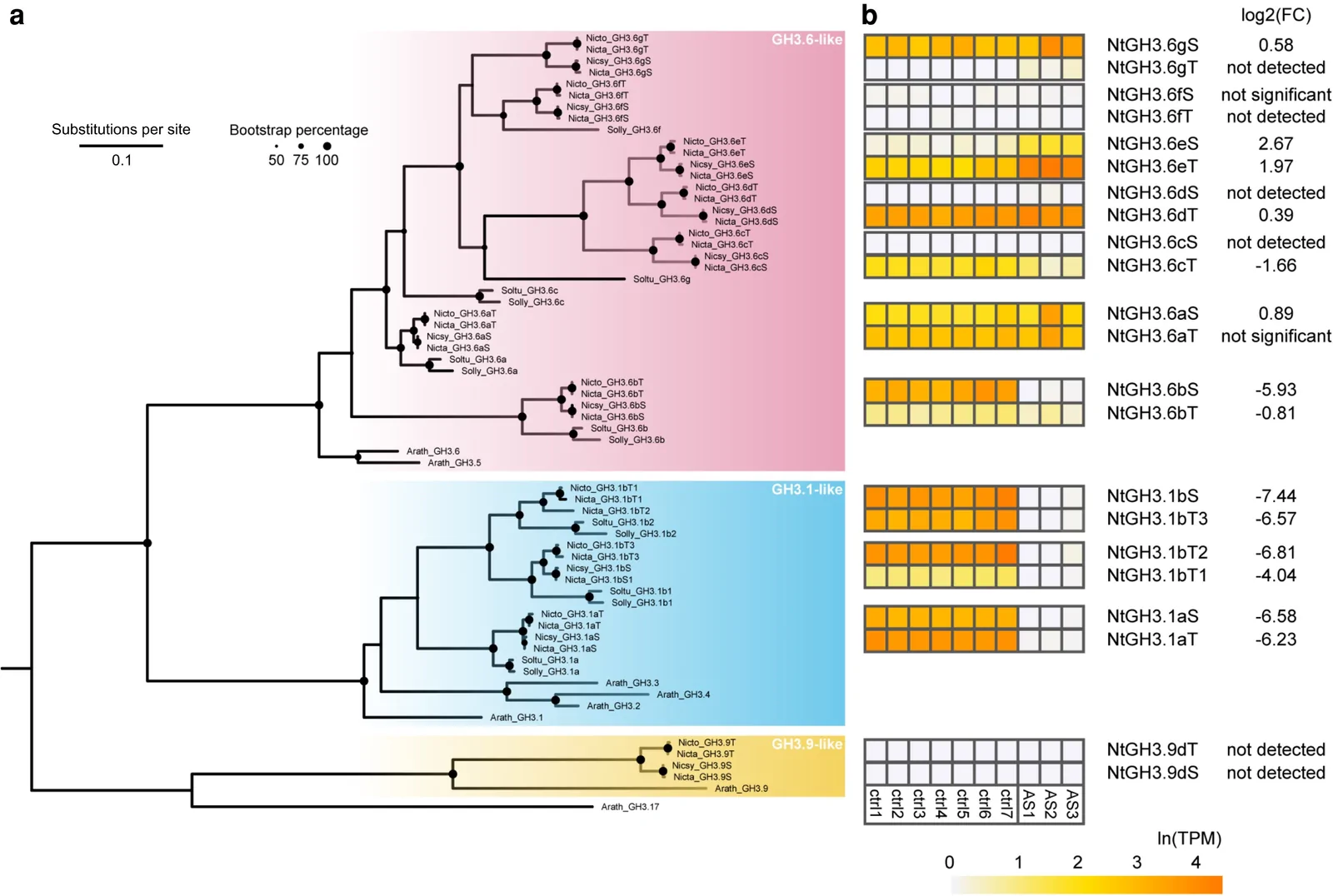
Phylogenetic analysis and transcript levels of GH3s. (a) Maximum likelihood tree of auxin-related GH3s from *Arabidopsis thaliana* (Arath), *Nicotiana tabacum* (Nicta), *Nicotiana sylvestris* (Nicsy), *Nicotiana tomentosiformis* (Nicto), *Solanum lycopersicum* (Soly) and *Solanum tuberosum* (Sotu). The phylogenetic tree was created using the LG evolutionary model in PhyML algorithm and validated by 500 bootstrap replicates. Full accession numbers and the analyzed multiple sequence alignment (MSA) tool are listed in Table S1. (b) Transcript levels (in TPM) and fold-change values for *GH3* homologs in 2-day-old control (containing 2,4-D) and auxin-starved BY-2 cells (AS), determined by RNA-seq analysis (Müller et al. 2021).

Based on available genomic data for *Nicotiana tabacum* (Sierro et al. 2014, 2024; Edwards et al. 2017), and using RNA-seq data from our previous work (Müller et al. 2021), we identified 22 paralogues of *GH3* genes belonging to group II (Staswick et al. 2005) and analyzed their transcription in tobacco BY-2 cells cultured in the presence or absence of the synthetic auxin 2,4-dichlorophenoxyacetic acid (2,4-D; Fig. 1). Phylogenetic analysis of protein sequences from representative plant species showed that Group II GH3s form 3 distinct subgroups: GH3.1-like, GH3.6-like, and GH3.9-like, based on the *Arabidopsis thaliana* homologs AtGH3.1, AtGH3.5/6, and AtGH3.9 at the base of each subgroup (Fig. 1a). Gene symbols were assigned based on the closest homologs in *Arabidopsis thaliana* (Table S1). The tobacco *GH3* genes were delineated with letters a to g in an ascending order according to their phylogenetic distance from the Arabidopsis homolog at the base of the group. The designation S or T was chosen for assignment to the descendants of the species *Nicotiana sylvestris* or *Nicotiana tomentosiformis*, respectively, that are parental to *Nicotiana tabacum*. A subgroup of genes related to *GH3.1*-like showed high transcription in controls, whereas in auxin-starved (AS) cells, the transcription dropped to levels close to zero (Fig. 1b). In contrast, the majority of genes associated with the *GH3.6*-like subgroup exhibited relatively high expression in the absence of auxin (Fig. 1b), and they were both slightly downregulated or upregulated in auxin-supplied cells. We did not detect any transcriptional activity for genes from the *GH3.9*-like subgroup in BY-2 cells (Fig. 1b). Additionally, no *AtGH3.17* homologs were identified in *N. tabacum*.

### GH3.1- and GH3.6-like show different amino acid substrate preference

Based on their contrasting transcription under auxin-starved conditions, we selected 1 candidate gene from each subgroup for functional characterization and investigated the substrate preferences of these genes for amino acid conjugation. From the GH3.1-like subgroup, *NtGH3.1aT* was chosen because it exhibited the strongest expression under 2,4-D–supplemented conditions and it represents the closest tobacco ortholog of the well-characterized Arabidopsis GH3.1-like enzymes. From the GH3.6-like subgroup, *NtGH3.6eT* was selected due to its distinctive expression pattern showing a high level of expression in auxin-starved conditions (Fig. 1b). We generated the BY-2 lines XVE::NtGH3.1aT-GFP and XVE::NtGH3.6eT-GFP for β-estradiol (EST)-inducible overexpression (Zuo et al. 2000), and knock-out lines by using CRISPR/Cas9-mediated gene editing (crNtGH3.1a and crNtGH3.6e). Lines for inducible expression of GH3 showed substantially increased expression of target genes, as validated by RT-qPCR (Figure S1), while knocked-out lines were verified by sequencing of the targeted regions (Figures S2, S3; Table S2).

**Figure 2:**
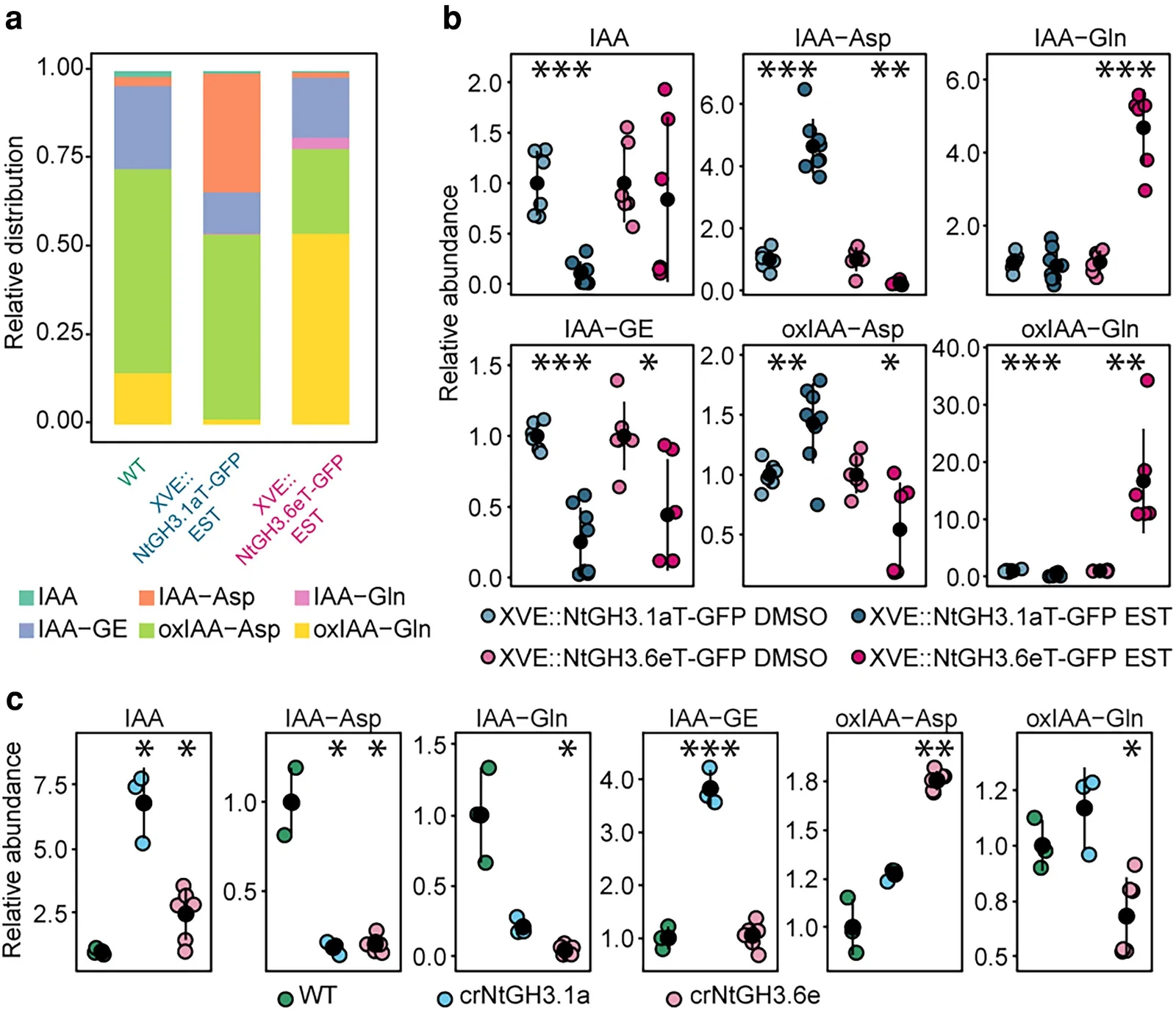
IAA metabolic profiles in BY-2 cells with modified expression of NtGH3.1aT and NtGH3.6eT. Relative distribution of IAA metabolites (a) and their abundance (b) in control (WT) and β-estradiol (EST)-induced overexpression lines XVE::NtGH3.1aT-GFP and XVE::NtGH3.6eT-GFP. Values are expressed relative to DMSO-treated cells (b). (c) Relative abundance of IAA metabolites in knocked-out lines crNtGH3.1a and crNtGH3.6e. Values are expressed relative to WT cells. 1 µM IAA was applied to 2-day-old cells for 4 h (a, b) or 2 h (c). For each experiment, 1 to 3 independent lines were used, and 3 to 5 biological replicates per line were performed. Data points and error bars indicating mean ± SE are shown. b and c, Student's t test *P*-values: *P < 0.05, **P < 0.01, ***P < 0.001.

To compare auxin metabolism in knocked-out and overexpressed lines, we performed liquid chromatography-tandem mass spectrometry (LCMS) IAA metabolite profiling after application of 1 µM IAA to 2-day-old cells. 2-Oxindole-3-acetyl-aspartate (oxIAA-Asp), IAA-GE, indole-3-acetyl-aspartate (IAA-Asp), and oxIAA-Gln were the most abundant conjugates detected in control lines and lines overexpressing NtGH3.1aT and NtGH3.6eT; however, the relative representation of individual metabolites differed (Fig. 2a). We evaluated concentrations of IAA-AA intermediates and the subsequent oxIAA-AA metabolites, products of DAO1 activity (Hayashi et al. 2021; Müller et al. 2021). Because DAO1 does not act on IAA-GE (Hayashi et al. 2021), IAA-GE is considered a terminal metabolite. In induced XVE::NtGH3.1aT-GFP cells, we observed significantly elevated levels of IAA-Asp and oxIAA-Asp compared to noninduced controls, accompanied by a reduction of free IAA and IAA-GE (Fig. 2b). In contrast, induced XVE::NtGH3.6eT-GFP cells accumulated significantly higher levels of IAA-Gln and oxIAA-Gln, for which the latter displayed more than 15-fold higher levels compared to the noninduced control, while IAA-Asp, IAA-GE, and oxIAA-Asp levels were lower.

Further evidence supporting isoform-specific amino acid substrate preferences was observed in lines carrying the mutant forms of *NtGH3.1a* or *NtGH3.6e* genes. In crNtGH3.1a cells, a significant increase in free IAA and IAA-GE was measured, along with a decrease in IAA-Asp and IAA-Gln (Fig. 2c). Conversely, crNtGH3.6e cells contained higher levels of free IAA and oxIAA-Asp, but reduced levels of IAA-Asp, IAA-Gln, and oxIAA-Gln. All metabolite measurements in mutated lines were compared with those in wild-type (WT) cells. These results support different substrate preferences between the 2 GH3 phylogenetic subgroups: the selected homolog of NtGH3.1a shows a preference toward Asp, whereas the selected homolog of NtGH3.6e shows preference toward Gln. To validate GH3-mediated conjugation of Asp and Gln to IAA, we performed a bacterial assay using NtGH3.1aT- and NtGH3.6eT-expressing *Escherichia coli* cultures, and GFP-expressing bacteria were used as a negative control. In NtGH3.1aT*-*expressing cultures, levels of IAA-Asp and IAA-Gln were 3-fold and 3.5-fold higher, respectively, compared to the GFP-expressing controls in the presence of IAA and the corresponding amino acid substrate (Figure S4). NtGH3.6eT*-*expressing bacteria showed approximately 250-fold and over 1,000-fold increases in IAA-Asp and IAA-Gln levels, respectively.

Together, our results demonstrate that both tested GH3 forms mediated the conjugation of AAs to IAA, but with a differential affinity toward distinct substrates correlating with their phylogenetic classification. The GH3.1-like representative (NtGH3.1aT) showed substantially higher affinity toward Asp, whereas the GH3.6-like representative (NtGH3.6eT) displayed greater affinity toward Gln, contributing to the formation of the recently identified metabolite oxIAA-Gln (Dobrev et al. 2023). Nevertheless, both enzymes demonstrated a certain degree of substrate promiscuity that does not occur in vivo in BY-2 cells.

### Substrate preference is based on corresponding subgroup

**Figure 3:**
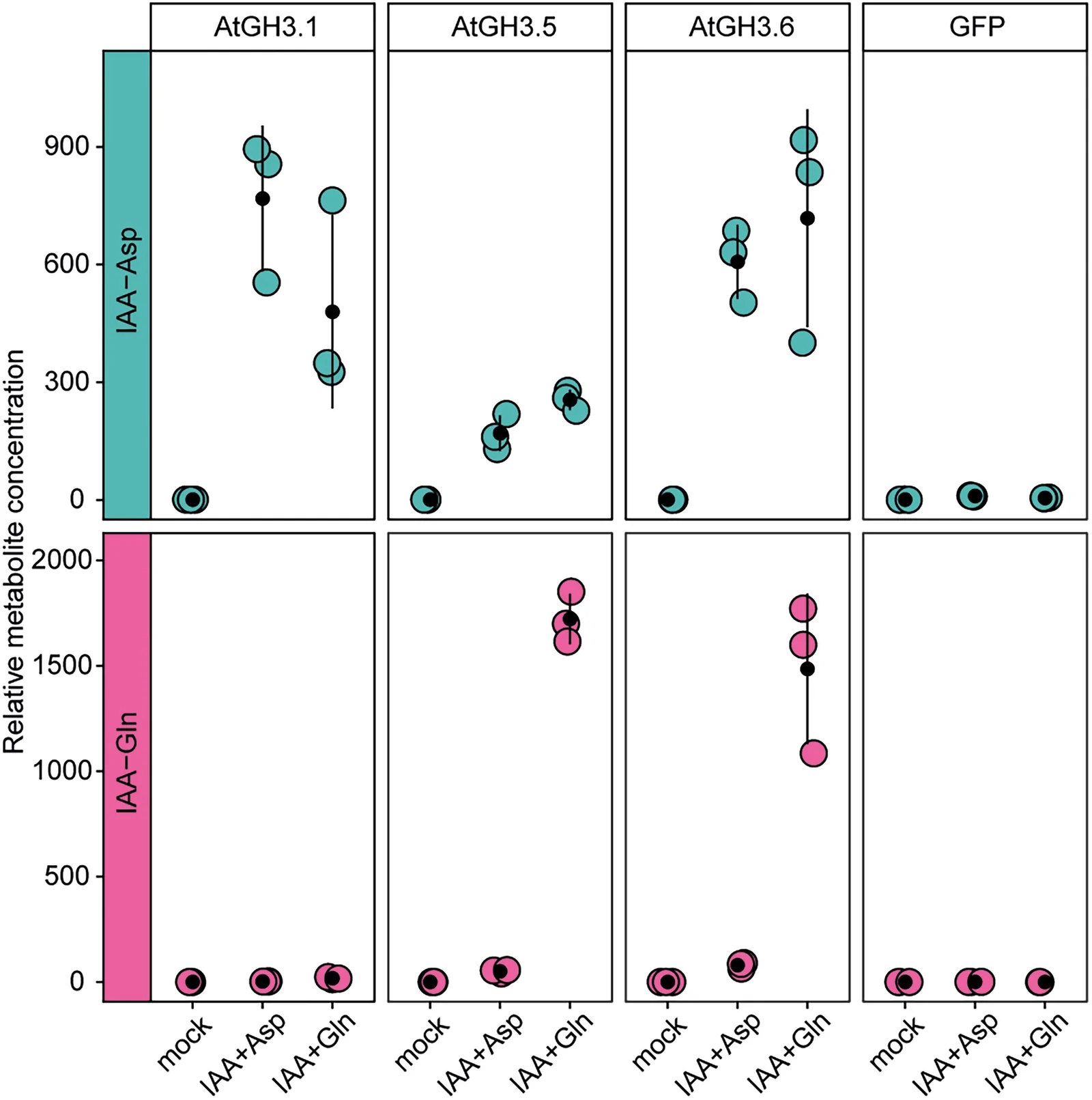
Levels of IAA-Asp and IAA-Gln in cell lysate of *Arabidopsis thaliana* GH3-expressing bacterial cultures supplemented with IAA and Asp or IAA and Gln. Arabidopsis homologs, AtGH3.1 from the GH3.1-like subgroup, AtGH3.5 and AtGH3.6 from GH3.6-like subgroup. Values are related to the GFP-expressing bacterial culture, which was used as a negative control. Three biological replications were performed. Data points and error bars indicating mean ± SE are shown.

To assess whether GH3 subgroup affinity toward Asp or Gln also occurs in Arabidopsis, we performed a bacterial assay of representative Arabidopsis homologs for both group II clades, homolog AtGH3.1 (GH3.1*-*like), and 2 homologs AtGH3.5, and AtGH3.6 (GH3.6*-*like). Samples collected from AtGH3.5- and AtGH3.6-expressing bacteria exhibited 1,700-fold and 1,500-fold higher levels of IAA-Gln after feeding with Gln, and 160-fold and 600-fold higher levels of IAA-Asp after feeding with Asp, respectively, compared to GFP-expressing controls (Fig. 3). Additionally, in samples from AtGH3.1*-*expressing bacteria, levels of IAA-Asp and IAA-Gln were approximately 900-fold and 20-fold higher, respectively, compared to controls. These results corroborate our findings for tobacco GH3 enzymes, confirming that the GH3.6-like subgroup exhibits a higher activity toward Gln, whereas the GH3.1-like subgroup shows a strong preference toward Asp. Notably, this Gln-specific activity is a conserved feature of the entire GH3.6-like subgroup in Arabidopsis, which comprises only 2 members, both of which display a remarkable affinity for Gln.

### NtGH3s are localized in the nucleus and cytoplasm

**Figure 4:**
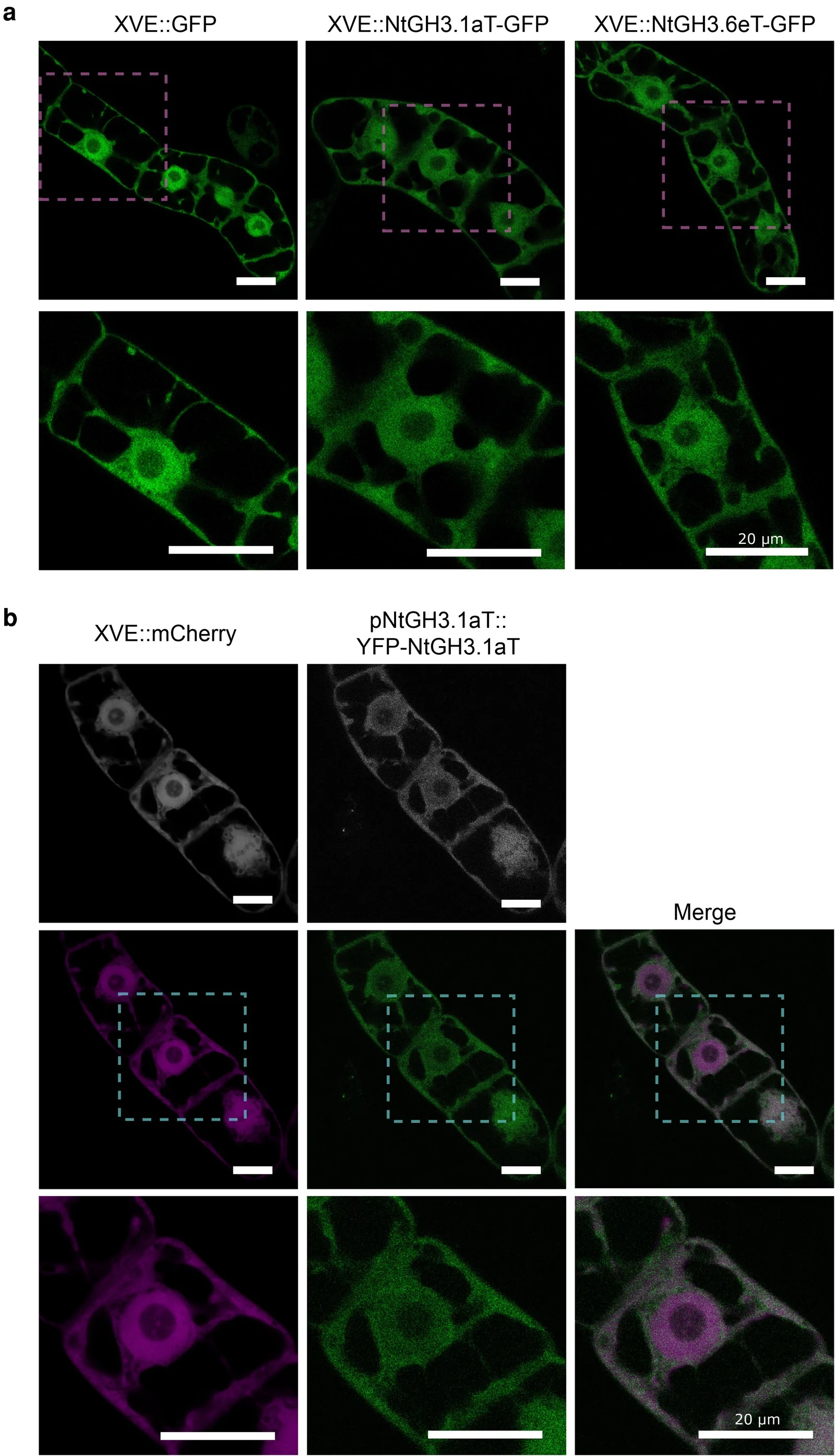
Nuclear and cytoplasmic localization of GH3 proteins in tobacco BY-2 cells. (a) Confocal images of 2-day-old BY-2 cells expressing control XVE::GFP (left column), XVE::NtGH3.1aT-GFP (middle column), and NtGH3.6eT-GFP (right column), all under β-estradiol (EST)-inducible promoter. Magnification of the rectangle areas (upper row) are shown in lower row. (b) Confocal images of 2-day-old BY-2 cells expressing XVE::mCherry&pNtGH3.1aT::YFP-NtGH3.1aT; control XVE::mCherry (in magenta) under EST-promoter (left column), pNtGH3.1aT::YFP-NtGH3.1aT (in green) under native promoter (middle column), and merged image of XVE::mCherry and pNtGH3.1aT::YFP-NtGH3.1aT (right column). Magnifications of the rectangular areas (middle row) are shown in lower row. Scale bars for all images from both panels = 20 µm.

**Figure 5:**
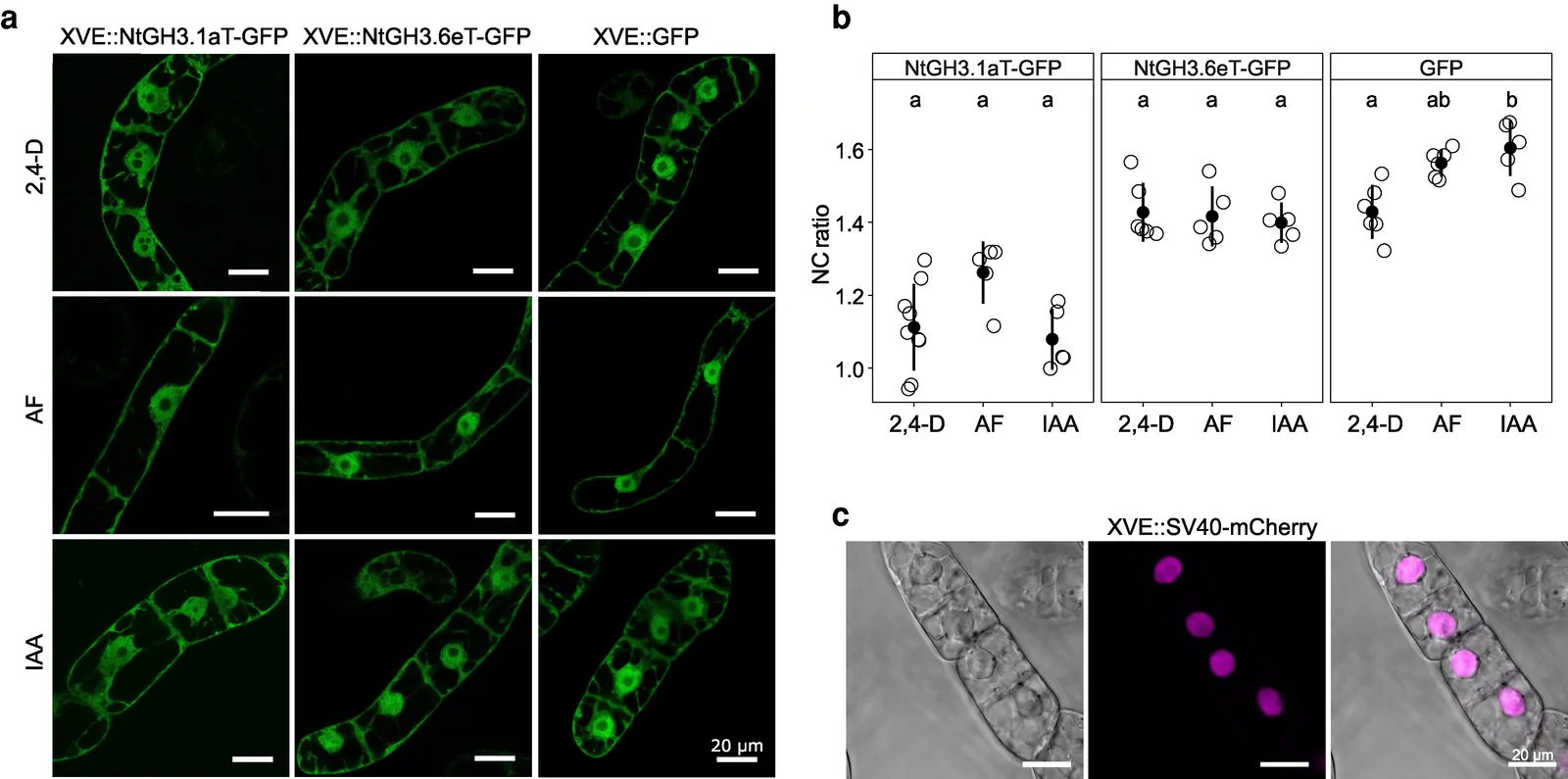
Effect of auxin availability on subcellular localization of GH3s and quantification of nuclear/cytoplasmic ratio of GFP signal. (a) Confocal images of 2-day-old cells expressing XVE::GFP, XVE::NtGH3.1aT-GFP, and XVE::NtGH3.6eT-GFP, all under β-estradiol-inducible promoter with or without the presence of auxin for 48 h. The upper row illustrates cells cultured in control medium with 1 µM 2,4-D (corresponding to those shown in Fig. 4a); the middle row in auxin-free (AF) medium; the lower row in medium with 1 µM IAA. (b) GFP signal from cells of inducibly overexpressing lines XVE::NtGH3.1aT-GFP, XVE::NtGH3.6eT-GFP, and XVE::GFP was quantified as a mean of all nuclear regions of interest (ROI) divided by the mean of all cytoplasmic ROIs per 1 cell. Data points represent a mean nuclear/cytoplasmic (NC) ratio of 4 to 12 cells. For each measurement, 2 to 4 independent lines were used, and 2 to 3 biological replicates per line were performed. Differences between auxin conditions were determined using the Kruskal-Wallis test followed by post-hoc tests (Dunn-Bonferroni correction). A *P*-value threshold of 0.05 was adopted. Data points and error bars indicating mean ± SE are shown. (c) Confocal images of 2-day-old cells expressing a marker for nuclear localization XVE::SV40-mCherry (in magenta). Scale bars for all images (a, c) = 20 µm.

Enzymatic reactions involved in auxin metabolism have been found in different cellular compartments, as reviewed by Casanova-Sáez et al. (2021). To determine whether the selected GH3 isoforms exhibit different subcellular localization, we used our lines expressing C-terminal GFP translational fusions of NtGH3.1aT and NtGH3.6eT coding sequences (CDS), driven by an EST-inducible promoter (Zuo et al. 2000). A GFP signal was observed in both the cytosol and nucleus for both tested GH3 proteins (Fig. 4a). We noticed a substantially lower nucleus/cytoplasm ratio for XVE::NtGH3.1aT-GFP compared to XVE::NtGH3.6eT-GFP and free GFP (Fig. 5b), potentially suggesting some specific nuclear import and export mechanisms for both GH3 proteins. To exclude the presence of free GFP, which could diffuse freely into nuclei, we performed anti-GFP Western blotting on protein extracts from all tested lines. We detected a strong band with a size of 100 kDa corresponding to intact GH3-GFP and no or a very faint band corresponding to free GFP (Figure S5), confirming the integrity of the GFP fusion proteins and the dual cytosolic and nuclear localization of both GH3 forms.

To test whether the subcellular localization of GH3 s might reflect the overall amount and availability of auxin, we analyzed the fluorescence of XVE::NtGH3.1aT-GFP and XVE::NtGH3.6eT-GFP cells grown in the presence of auxin (IAA or 2,4-D) or its absence (auxin-free, AF) for 48 hours. We observed GFP fluorescence in both cytosol and the nucleus under all conditions (2,4-D, IAA, and AF; Fig. 5a), indicating that the localization of NtGH3.1aT and NtGH3.6eT is not affected by auxin availability. The distribution of NtGH3.1aT and NtGH3.6eT between the nucleus and cytoplasm, quantified as a nuclear/cytoplasmic ratio of intensity mean value, was approximately 1.2 to 1.4 in all analyzed samples (Fig. 5b). As a control, we used BY-2 cells expressing free GFP under an EST-inducible promoter, which showed a stable nuclear/cytoplasmic ratio of intensity value of approximately 1.5, regardless of auxin (Fig. 5b). For nucleus recognition, the marker line XVE::SV40-mCherry with a viral nuclear localization signal (NLS) was employed (Fig. 5c).

Subcellular localization of GH3 proteins under native promoters *in planta* remains largely unexplored and poses several challenges. The *GH3* genes typically exhibit low expression, rapid turnover, and context-specific roles, such as in differentiation of the lateral root (Wang et al. 2023) or regulation of the primary root meristem size (Di Mambro et al. 2017; Pierdonati et al. 2019), often with tissue- or cell-specific expression patterns that complicate localization studies. To address these observations, we used tobacco BY-2 cells, allowing analysis simulating the single-cell level. We generated BY-2 lines expressing NtGH3.1aT-GFP under its respective native promoter and terminator (pNtGH3.1aT::NtGH3.1aT-GFP). Despite multiple attempts, we were unable to clone the promoter and terminator sequences of *NtGH3.6eT*; thus, we further focused our native expression analysis on NtGH3.1aT. Interestingly, no detectable GFP signal was observed in pNtGH3.1aT::NtGH3.1aT-GFP lines (Figure S6), raising questions regarding the strength or expression potency of the *NtGH3.1aT* native promoter. However, its functionality was positively tested in a reporter line expressing GFP under the control of the *NtGH3.1aT* promoter and terminator (Figure S7). To overcome the lack of detectable expression from the native promoter in the C-terminal GFP fusion, we generated an N-terminal yellow fluorescent protein (YFP) fusion construct, with XVE::mCherry as a nuclear and cytoplasmic marker, (XVE::mCherry&pNtGH3.1aT::YFP-NtGH3.1aT), which, upon transformation, confirmed clear cytoplasmic and nuclear localization of the protein product (Fig. 4b). These results indicate that N-terminal fusion is an important factor when designing natively regulated expression of GH3 proteins, suggesting a potential issue at the promoter-CDS interface or with start codon recognition within NtGH3.1aT CDS.

Overall, our localization data suggest that GH3 proteins are localized in both the nucleus and the cytoplasm when driven by both inducible and native promoters. This localization does not primarily reflect the availability of auxin from the extracellular space.

## Discussion

The *GH3* gene family encodes enzymes that conjugate plant hormones with amino acids. We previously demonstrated that not all *Nicotiana tabacum GH3* genes are activated by auxin, (Müller et al. 2021). Our phylogenetic analysis identified 22 *NtGH3* genes, classified into 3 subgroups: *GH3.1*-like, *GH3.6*-like, and *GH3.9*-like (Fig. 1). In a study examining GH3s across various species, group II has been further subdivided into 4 subsets based on phylogenetic relationships and synteny: IIA1 and IIA2, comprising *AtGH3.1-4* and IIB1 and IIB2, which include *AtGH3.5* and *AtGH3.6* (Okrent and Wildermuth 2011). These classifications are consistent with our findings. Notably, *AtGH3.9* and *AtGH3.17* were assigned to group IIIA, despite their typical classification as auxin-related members of group II (Staswick et al. 2005). Analyses presented in this work focused on functional differences between representative protein candidates corresponding to the GH3.1-like and GH3.6*-*like subgroups.

oxIAA-Asp The final product of the IAA-Asp conjugation pathway is oxIAA-Asp, formed via DAO1 activity (Hayashi et al. 2021; Müller et al. 2021). The GH3 substrate preference was assessed using IAA-AA intermediates and DAO1-derived oxIAA-AA products. Direct oxIAA conjugation with AAs occurs but contributes little to oxIAA-Asp accumulation compared with IAA-Asp oxidation in Arabidopsis (Brunoni et al. 2023). In tobacco BY-2 cells, oxIAA conjugation was observed only with Gln and at low frequency (Dobrev et al. 2023). Recently, the metabolite oxIAA-Gln, derived from IAA-Gln, was identified in a non-peer-reviewed report (Dobrev et al. 2023), and we identified Gln as the preferred substrate for NtGH3.6eT and Asp as the preferred substrate for NtGH3.1aT through three independent experiments: overexpression lines, knock-out mutant lines, and bacterial assays. NtGH3.1aT clusters with GH3.1-like members such as AtGH3.2/3/4 and VvGH3.1, which prefer Asp (Staswick et al. 2005; Peat et al. 2012; Brunoni et al. 2019, 2023; Široká et al. 2025). Notably, while NtGH3.6eT exhibited low affinity for Asp (Fig. 2b, c), Arabidopsis GH3.6*-*like enzymes have been shown to strongly favor Asp (Staswick et al. 2005; Westfall et al. 2016; Brunoni et al. 2019, 2023; Široká et al. 2025). On the other hand, AtGH3.5/6 recognized various amino acid substrates, similar to VvGH3.6, which conjugates a broad range of AAs, including Gln and Asp (Staswick et al. 2005; Böttcher et al. 2012), but with a preference for Gln, consistent with our findings (Fig. 3). NtGH3.6eT showed a clear preference for Gln; however, although tobacco GH3s displayed broader substrate promiscuity in the bacterial system (Figure S4), this was not fully reflected in vivo, particularly in BY-2 overexpressor lines (Fig. 2b). Additionally, Gln conjugation has also been reported for IBA and JA by AtGH3.15 (Sherp et al. 2018). Despite this, IAA-Gln and oxIAA-Gln remained a rarely reported metabolites, possibly due to rapid turnover and low accumulation *in planta*. IAA-Gln has been detected in heterologous systems and occasionally in Arabidopsis (Barratt et al. 1999). Similar findings were reported for IAA-Leu and IAA-Ala, which accumulate in *ilr/ill* triple mutants, but remain low in wild-type plants due to ILR1-mediated hydrolysis (Rampey et al. 2004). These observations suggest functional diversity among IAA conjugates, with negatively charged forms, such as IAA-Asp, potentially serving as long-term storage for IAA. In contrast, polar forms, such as IAA-Gln, may act as short-term, readily accessible auxin reservoirs. This functional diversification would allow plants to fine-tune auxin homeostasis across different temporal scales—rapid responses mediated by Gln conjugation and sustained regulation through Asp conjugation. Furthermore, IAA-AA conjugation may primarily function as an AA reservoir rather than an IAA source, regarding the importance of Asp in plant metabolism (Han et al. 2021). The evolutionary conservation of these substrate preferences across species (tobacco and Arabidopsis in this study; *Vitis vinifera*, Böttcher et al. 2012) suggests the presence of fundamental regulatory mechanisms rather than species-specific adaptations.

Tobacco BY-2 cells provide an ideal single-cell model for dissecting intracellular auxin metabolism. Unlike plants, this cell-autonomous system eliminates tissue-level complexity and long-distance transport variables, which confound the interpretation of enzyme-specific activities. Crucially, metabolic profiling of these lines serves as a robust in vivo competition assay: while the exogenous input was limited to IAA, the GH3 enzymes selected their preferred substrates (Asp or Gln) from the complex endogenous AA pool available within the cell. Unlike Arabidopsis, where esters such as IAA-GE and oxIAA-GE dominate auxin metabolism (Mateo-Bonmatí et al. 2021; Casanova-Sáez et al. 2022; Hladík et al. 2023; Škyvarová et al. 2025), *Nicotiana tabacum* cells accumulate higher levels of amino acid conjugates (Dobrev et al. 2023). IAA metabolic profiling in BY-2 cells reveals a substantial accumulation of auxin decarboxylation-derived metabolites, a pathway recently identified as an important component of IAA metabolism in both BY-2 cells and other plants, including Arabidopsis (Dobrev et al. 2023). In addition to substrate preferences, metabolic profiling indicated significant alterations in auxin conjugates. Loss of the major Asp-conjugating enzyme NtGH3.1aT (crNtGH3.1a) resulted in elevated free IAA levels, highlighting its key role in IAA homeostasis. IAA-GE, but not IAA-Gln, accumulated as an alternative conjugate. Simultaneously, overexpression of NtGH3.1aT (XVE::NtGH3.1aT-GFP) depleted IAA-GE levels (Fig. 2b, c), suggesting competition between GH3-mediated Asp conjugation and UGT-mediated glucose conjugation. This is consistent with previous findings in Arabidopsis, where overexpression of UGT84B1 increased IAA-GE at the expense of IAA-Asp (Jackson et al. 2002; Aoi et al. 2020; Mateo-Bonmatí et al. 2021), supporting the notion that GH3s and UGTs act as alternative pathways modulating free IAA levels. In contrast, NtGH3.6eT knockout (crNtGH3.6e) led to oxIAA-Asp accumulation, not IAA-GE, and its overexpression (XVE::NtGH3.6eT-GFP) slightly reduced both IAA-Asp and IAA-GE levels (Fig. 2b, c), suggesting that Gln conjugation operates independently, with distinct regulatory and functional roles. The consistent pattern observed across three independent experimental systems (overexpression lines, knockout mutants, and bacterial heterologous expression) as presented here, and the concordance with published studies (Staswick et al. 2005; Peat et al. 2012; Brunoni et al. 2023) provide convincing proof of the massive complexity of IAA metabolism.

Early analyses of GH3 amino acid sequences predicted cytoplasmic localization (Liu et al. 2005), a finding later confirmed by sucrose density gradient fractionation, which identified AtGH3.17 in the cytoplasm without membrane association (Di Mambro et al. 2019). Studies using artificial promoters further supported cytoplasmic or dual cytoplasmic-nuclear localization for several GH3s across species: PpGH3.1 in *P. patens* protoplasts (Ludwig-Müller et al. 2009), AtGH3.3 and AtGH3.5 in Arabidopsis roots (Liu et al. 2025; Široká et al. 2025), OsGH3.5 in rice protoplasts (Zhang et al. 2015). In this study, we provide a comparative analysis of the subcellular localization of GH3 proteins with distinct substrate preferences and show their cytoplasmic and nuclear presence (Fig. 4). The NC ratio did not differ substantially, when cells were treated with external auxins or incubated in auxin-free media (Fig. 5). Additionally, we generated BY-2 lines expressing pNtGH3.1aT::YFP-NtGH3.1aT under its native regulatory elements, which confirmed consistent nuclear and cytoplasmic localization of NtGH3.1aT protein (Fig. 4b), and validated the fact that dual nuclear-cytoplasmic distribution is not an artifact of protein overexpression due to strong artificial promoters, which can saturate nuclear transport or retention mechanisms and cause mistargeting (Tanz et al. 2013). These results imply that NtGH3 subcellular localization is independent of both auxin availability and substrate preference, with members of different GH3 subgroups consistently localizing to both cytoplasm and nucleus. Subcellular compartmentalization of auxin metabolism highlights the need for precise, compartment-specific control of free IAA. Our data, together with prior work on DAO1 (Müller et al. 2021), indicate that the 2 principal IAA-inactivation pathways, amino acid conjugation by GH3s and irreversible oxidation by DAO1, operate in both cytoplasm and nucleus, whereas IAA-amido hydrolases (ILR1/ILLs) localize predominantly to the endoplasmic reticulum (Sanchez Carranza et al. 2016; Zhang et al. 2016). This topology is well poised to tune the 2 major arms of auxin signaling that unfold on distinct time scales and in distinct compartments: rapid, non-transcriptional responses initiated at the cell surface and in the cytoplasm versus the canonical TIR1/AFB-AUX/IAA-ARF transcriptional response confined to the nucleus (reviewed in Vanneste et al. 2025). In this framework, cytoplasmic GH3 activity can buffer transient stimulus-evoked increases in cytoplasmic auxin levels, thereby shaping the amplitude and duration of fast outputs such as membrane depolarization, Ca^2+^ influx, and rapid growth inhibition, while nuclear GH3 activity sets the instantaneous occupancy of TIR1/AFB co-receptors and imposes a local, auxin-inducible negative feedback on transcriptional outputs. The dual localization of DAO1 further sharpens this compartmental control by providing an irreversible sink in both spaces, whereas ER-localized ILR1/ILLs may selectively re-supply auxin from conjugates at ER-nucleus and ER-plasma membrane interfaces.

IAA conjugates represent a notable class of metabolites, typically facilitating reduction of free IAA to approximately 1% of total auxin content. Unlike free IAA and AAs, both actively transported via specific carriers, IAA conjugates appear largely immobile, possibly passively diffusing (Hangarter et al. 1980). Their apparent lack of transport may reflect a biological function: to prevent loss or degradation by retaining them intracellularly in a conjugated, inactive form. This storage mechanism could serve roles in both auxin signaling and nitrogen metabolism, as it is proposed in root hair formation (Montiel and Dubrovsky 2024). IAA-AA conjugates are also chemically more stable than free IAA (Hangarter et al. 1980). Conjugation-mediated storage of IAA is only effective when separated from DAO1 activity, as ILR1-like hydrolases can cleave even oxidized conjugates (Hayashi et al. 2021), but oxidation of IAA-AA conjugates does not affect AA part of the molecules. Despite their similar subcellular localization, the mechanisms of their interaction remain unclear.

## Materials and methods

### Plant material

Tobacco BY-2 cell line (*Nicotiana tabacum* L. cv Bright Yellow 2; Nagata et al. 1992) was maintained in liquid Murashige and Skoog (MS) medium (3% sucrose (w/v), 4.3 g/L MS salts, 100 mg/L myo-inositol, 1 mg/L thiamine, 0.2 mg/L 2,4-D, and 200 mg/L KH_2_PO_4_; pH 5.8), in the dark, at 27 °C, under continuous shaking (150 rpm; orbital diameter 30 mm). Suspension cultures were sub-cultured weekly. Transgenic cells and calli were maintained on the same medium supplemented with 100 µg/mL cefotaxime and 20 µg/mL hygromycin. Medium for calli was solidified with 6 g/mL agar. Unless stated otherwise, all chemicals and kits were obtained from Merck.

### Phylogenetic analyses, gene and protein annotation

For the identification of *Nicotiana tabacum*, *Nicotiana sylvestris,* and *Nicotiana tomentosiformis* paralogues of *Arabidopsis thaliana* GH3.1, GH3.5, and GH3.6, we used BLAST tool (Altschul et al. 1990) against the genomes of *N. tabacum*, *N. sylvestris,* and *N. tomentosiformis* available at NCBI and Sol Genomics datasets of cultivars K326, TN90, and v1.0 (Sierro et al. 2014, 2024; Edwards et al. 2017). The identified protein sequences were cross-validated across the individual *N. tabacum* datasets to pinpoint potential errors, such as amino acid insertions or deletions, introduced by automatic exon-intron annotation of the deposited genomes. In the case of a putatively incorrectly annotated protein sequence, we reconstructed the sequence using the FGENESH + gene prediction tool with the closest GH3 paralogue amino acid sequence and the respective genomic locus nucleotide sequence as inputs. For *Arabidopsis thaliana*, *Solanum tuberosum* and *Solanum lycopersicum*, we extracted the previously published Group II representative sequences (Zhang et al. 2018) from the Phytozome public database (Goodstein et al. 2012). The corrected protein dataset was then aligned using the CLUSTAL multiple sequence alignment (MSA) tool in the Seaview software (Galtier et al. 1996). Low conservation regions were trimmed from the resulting MSA by the GBlocks algorithm. The trimmed MSA was then used as an input for maximum-likelihood tree construction by PhyML and validated by 500 parametric bootstrap replicates. The phylogenetic tree was graphically edited in Figtree. Based on the phylogenetic tree, we subsequently annotated the identified tobacco GH3 homologs. The GH3 subgroups were delineated with letters a-f in an ascending order according to their phylogenetic distance from the Arabidopsis homolog at the base of the group. The origin of genes from the paternal species *N. sylvestris* or *N. tomentosiformis* was distinguished by adding S or T letters at the end of the gene name. A summarizing table, including protein and transcript coding sequences, is shown in Table S1.

### Estimation of GH3 transcript levels

To assess transcript levels of the above-described reannotated GH3 forms, we reanalyzed our RNA-seq data deposited in the Gene Expression Omnibus under accession GSE160438. Similar to Müller et al. (2021), the transcript abundances (transcripts per million—TPM) were determined using Salmon (Patro et al. 2017) with the parameters –posBias, –seqBias, –gcBias, and –numBootstraps 30. Index was built from the CDS dataset of the recently released version of the *Nicotiana tabacum* cultivar K326 (Sierro et al. 2024), in which all GH3 homologs were replaced with manually reconstructed coding sequences (Table S1). Visualization of transcript abundances and determination of differential expression were done using the sleuth package in R (Pimentel et al. 2017). Transcripts with q-value ≤ 0.05. were considered to be significantly differentially expressed.

### Cloning of tobacco *GH3* genes, targeted mutagenesis, and transformation of BY-2 cells

The GoldenBraid 3.0 cloning system was used for the preparation of all plasmid constructs (Sarrion-Perdigones et al. 2013; Vazquez-Vilar et al. 2017). *GH3* CDSs were amplified from RNA isolated from BY-2 cells and reverse transcribed to cDNA, and the promoter and terminator regions of *NtGH3.1aT* from the genomic DNA of BY-2 (2,000 bps upstream from the start codon and 500 bps downstream from the stop codon, respectively) with specific primers (Table S3) designed using GB Domesticator tool (https://goldenbraidpro.com/). For target draft, CRISPRdirect tool was used (Naito et al. 2015), and for designing guideRNAs (gRNA), GB CRISPR Domesticator was employed (Vazquez-Vilar et al. 2016; Selma et al. 2019). All remaining DNA parts were obtained from GoldenBraid 2.0 kit from Diego Orzaez (Addgene kit # 1000000076) or MoClo kit from Sylvestre Marillonnet (Addgene kit # 1000000044). The restriction/ligation reactions were performed using Type IIS restriction enzymes *BsmBI* and *BsaI* (ThermoFischer Scientific) and T4 ligase (Promega).

gRNAs were designed to target both the S and T forms of *NtGH3.1a* and *NtGH3.6e* genes. To increase the chance for large deletions in the gene, 2 gRNAs were assembled for each gene with a distance between both sites of approximately 200 to 400 nucleotides (*NtGH3.1a* gRNA1: 5′-GCGTCTTTCTCACATGCAGGAGG-3′, gRNA2: 5′-GAGATCCAACGGATTGCTAACGG-3′; *NtGH3.6e*, gRNA1: 5′-TTGCTGTAATAATCAAGTATCGG-3′, gRNA2: 5′-GGACTCTACAGATATAGAGTCGG-3′). Therefore, each construct consisted of 2 gRNA-forming units to generate mutations in both forms S and T, with no or minimal homology to other *GH3* genes. Complete sequences of plasmid constructs are deposited on Zenodo (https://doi.org/10.5281/zenodo.18834594). To test CRISPR/Cas9-mediated mutation activity, genomic DNA was isolated from independent 3-week-old calli or 2-day-old cell suspension grown on selective medium and the corresponding region of genomic DNA was amplified by PCR using specific primers designed to recognize both S and T forms (Table S3). PCR fragments were ligated into the pJET 1.2 vector and sequenced. One and two lines with characterized knock-out inducing mutations in *NtGH3.1a* and *NtGH3.6e*, respectively, were selected for further experiments (Figures S2, S3; Table S2).

For GH3s localization experiments, GFP, YFP and mCherry were from MoClo Toolkit, from Nicola Patron (Addgene plasmid #50314, #50302, #50316; Engler et al. 2014), and nuclear localization signal (NLS) SV40 was also from MoClo kit, from Johannes Stuttmann (Addgene plasmid #105400; Gantner et al. 2018). Coding sequences (CDS) of *GH3* genes for overexpression were fused to their C-terminus with GFP CDS. All constructs were expressed under the β-estradiol-inducible XVE promoter. Approximately 2 to 4 transgenic lines were selected based on verification by fluorescence microscopy and RT-qPCR. For expression under a native promoter and terminator, YFP was fused to the N-terminus of *NtGH3.1aT* CDS.

For transformation of BY-2 cells, *Agrobacterium tumefaciens* strain GV2260 was used following a previously reported protocol (An et al. 1985), which was modified as described in (Klíma et al. 2019). After 4 to 6 weeks at solid selection MS media with hygromycin and cefotaxime, independent calli were harvested and tested for the presence of CRISPR/Cas9-generated mutations or for fluorescence signal. Suspensions established from verified calli were also tested for the presence of corresponding mutation or for the presence of a fluorescence signal.

### RNA isolation and quantitative reverse transcription PCR

Total RNA was isolated from 50–100 mg of 3-week-old BY-2 calli using the Plant Total RNA Mini kit (Favorgen) and treated with rDNase set (Macherey-Nagel) to eliminate contaminating DNA. The RNA concentration and purity were evaluated on Spectrophotometer DS-11+ (DeNovix) and on 0.8% agarose gels (v/w), respectively. Purified RNA was diluted 100 × and mixed with Master Mix GoTaq 1-Step RT-qPCR (Promega) and 500 nM primers designed to be specific to both S and T gene forms (Table S3). A 1-step real time qPCR was performed at 58 °C annealing temperature on a LightCycler480 instrument (Roche). As reference genes, tobacco aminopeptidase-like, fructokinase2, and ribokinase-like genes (GenBank accession numbers XM_016640300, KJ683760, and XR_001650512, respectively) were selected due to their stable expression in various conditions, previously published in (Müller et al. 2021). Relative transcript levels were calculated using the equation:


$$\mathrm{rel}.\mathrm{transcript}\;\mathrm{level}=\frac{\sqrt[{3}]{{\mathrm{eff}}_{\mathrm{ref}1}^{CP_{\mathrm{ref}1}}.{\mathrm{eff}}_{\mathrm{ref}2}^{CP_{\mathrm{ref}2}}.{\mathrm{eff}}_{\mathrm{ref}3}^{CP_{\mathrm{ref}3}}}}{{\mathrm{eff}}_{\mathrm{target}}^{CP_{\mathrm{target}}}}$$

where eff_ref_ and eff_target_ stand for the qPCR efficiencies of reference and target genes, and CP_ref_ and CP_target_ stand for the respective crossing points of reference and target genes. Positive transcript levels and the quality of PCR products were verified by melting curve analysis. Experiments were performed in 4 to 6 biological replicates, and 2 technical repetitions for each line.

### Metabolic analysis

For metabolic analysis, 10–30 mg of 2-day-old BY-2 cell suspension was used. Cells were harvested after 2 or 4 hours of incubation with 1 µM IAA. Inducible lines were treated 48 hours before adding IAA with 1 µM β-estradiol. Control suspensions were cultured with DMSO. Experiments were performed in 3 to 5 biological replicates and 2 technical repetitions for each line.

Extraction and purification of all IAA metabolites were performed according to Dobrev and Kamínek (2002); Dobrev et al. (2017, 2023). The metabolites were separated on Kinetex EVO C18 column (2.6 µm, 150 × 2.1 mm, Phenomenex). The mobile phases consisted of A) 5 mM ammonium acetate in water and B) 95/5 acetonitrile/water (v/v). The following gradient program was applied: 5% B (v/v) in 0 min, 7% B (v/v) in 0.1–5 min, 10 to 35% (v/v) in 5.1–12 min, 100% B (v/v) at 13–14 min, and 5% B (v/v) at 14.1 min. The LCMS system consisted of UHPLC 1290 Infinity II (Agilent Technologies) coupled to 6495 Triple Quadrupole mass spectrometer (Agilent Technologies). Data acquisition and processing were done with Mass Hunter software version B.08 (Agilent Technologies). The final relative value was calculated as the concentration of the metabolite divided by the sum of all IAA metabolites and then related to DMSO or WT controls.

### Cloning, protein production, and bacterial enzyme assay

For bacterial expression of tobacco *GH3* genes, coding sequences of *NtGH3.1aT* and *NtGH3.6eT* were cloned into the *BsaI* restriction site of a pETM-11 vector to express an N-terminally fused 6× His tag, as previously described (Široká et al. 2025). *Escherichia coli* BL21 (DE3) strains expressing recombinant AtGH3.1, AtGH3.5, and AtGH3.6, used in this work, were previously generated (Brunoni et al. 2019, 2023). Recombinant protein production and enzymatic assay of GH3 s were performed as described previously (Brunoni et al. 2023). Briefly, 500 µl of clarified cell lysate were incubated with 1 mM Asp or Gln, 3 mM ATP, 3 mM MgCl_2_, and 1 mM auxin substrate (IAA) for 5 h at 30 °C with constant shaking at 50 rpm in darkness. The assay was performed in three independent biological replicates, with each biological replicate measured in three technical replicates. Sample preparation and quantification of IAA-Asp and IAA-Gln were performed by LCMS as described above. Samples from GFP-producing bacterial culture were used as a negative control.

### Western blot analysis

Approximately 200 mg of liquid nitrogen frozen BY-2 suspension was homogenized and mixed with 2 mL of extraction buffer (150 mM HEPES; 150 mM NaCl; 10% glycerol; 10 mM EDTA, pH 7.5). Before each extraction, 10 mM DTT and 1% (v/v) NP-40 IGEPAL were added. Samples were centrifuged (15,000 g, 15 min, 4 °C) to separate the cell debris, and the supernatant was used for subsequent analyses. For measuring the concentration of protein content, Qubit Protein assay kit (Thermo Fisher) was used according to the manufacturer's instructions. A total of 20 µg of protein extracts was loaded for SDS-PAGE analyses in a 4%20% gradient Mini-PROTEAN TGX precast gels and transferred to PVDF membranes using TransBlot Turbo device (Bio-Rad). Western Blot was performed using monoclonal anti-GFP (1:1,000; Roche) primary and HRP-conjugated anti-mouse (1:5,000; Promega) secondary antibodies and developed using Azure Sapphire TM Biomolecular Imager (Azure Biosystems).

### Microscopy and image analysis

Fluorescence microscopy was performed using a Zeiss LSM 880 inverted confocal laser scanning microscope (Carl Zeiss GmbH, http://www.zeiss.com/) with a water 40× C-apochromatic objective (NA 1.2). GFP was excited with argon laser (488 nm), and emission detected at 495 to 552 nm, YFP was excited with argon laser (488 nm), and emission detected at 516 to 570 nm, and mCherry was excited with solid state laser (561 nm), and emission detected at 588 to 677 nm. Pinhole opening 0.73 to 0.93 AU, and pixel time 4.12 µs were used. The images of single optical sections through the area of the nucleus were analyzed using ZEN 2.6 software (Carl Zeiss GmbH, Germany). For nucleus recognition, differential interference contrast (DIC) images were used, original images are available on Zenodo (https://doi.org/10.5281/zenodo.18834594). The intensity mean values of the signal were quantified using interactively applied regions of interest (ROIs). ROIs of diameter 1,500 nm were positioned on the nucleus and cytoplasm areas, 4 ROIs for the nucleus and 4 ROIs for the cytoplasm per one cell, 50 to 54 cells were measured per sample. The final value was calculated as the mean of all nuclear ROIs related to the mean of all cytoplasmic ROIs per one cell. All images and measurements are deposited on Zenodo (https://doi.org/10.5281/zenodo.18834594). In experiments with auxin presence or absence in medium, cells were cultured for 48 hours with or without auxin. About 2 to 4 lines from each construct were used for all conditions, and 2 to 3 biological replicates per line were performed.

### Use of artificial intelligence

Claude Sonnet 4.6 and Claude Opus 4.6 (Anthropic) were used to assist with manuscript preparation. These tools were used for assistance in language & writing (improving grammar and language, paraphrasing and rewording, adjusting tone and style, translating text). We confirm that we had full oversight of all assisted work and confirm the validity of the related sections in the submission.

### Accession numbers

Sequence data from this article can be found in the GenBank/EMBL data libraries under accession numbers listed in Table S1.

## Supplementary Material

kiag412_Supplementary_Data

## Data Availability

The data that support the findings of this study are included as part of the article or as Supporting Information. The source data have been deposited on Zenodo (https://doi.org/10.5281/zenodo.18834594).
