# Supplementary material for: GH3 phylogenetic subgroups define divergent routes of auxin inactivation via aspartate and glutamine conjugation
Source: Plant Physiol. 2026 Jun 19;201(4):kiag412. doi: 10.1093/plphys/kiag412 (PMC13524089; doi:10.1093/plphys/kiag412)
Supplement: Supplementary file 1 [file kiag412_supplementary_data.zip › Supplementary data.pdf]

Supplementary figures

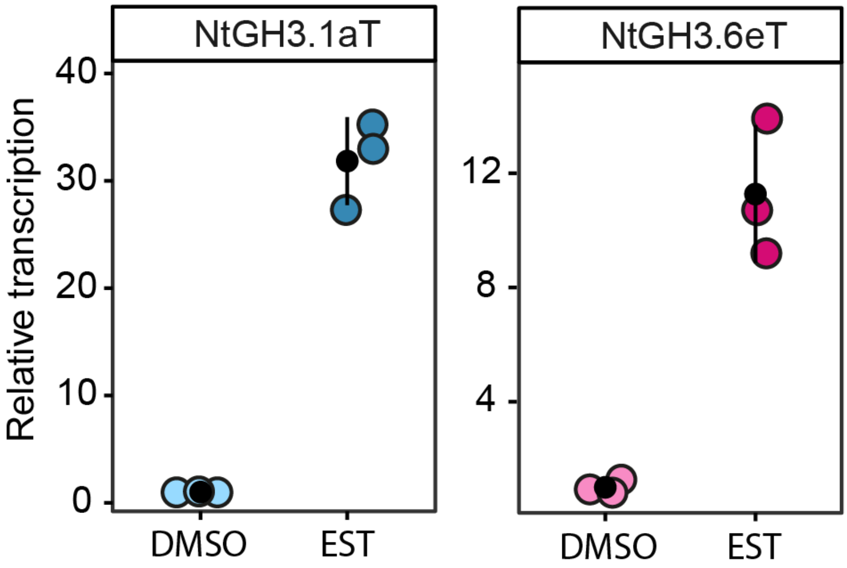

Supplementary Figure S1. RT-qPCR verification of overexpressing lines**.** The relative transcription of *NtGH3.1a and NtGH3.6e* genes in 2-day-old XVE::NtGH3.1aT-GFP and XVE::NtGH3.6eT-GFP cells. Values are related to non-induced (DMSO) controls. Three biological replications were performed. Data points and error bars indicating mean ± SE are shown.

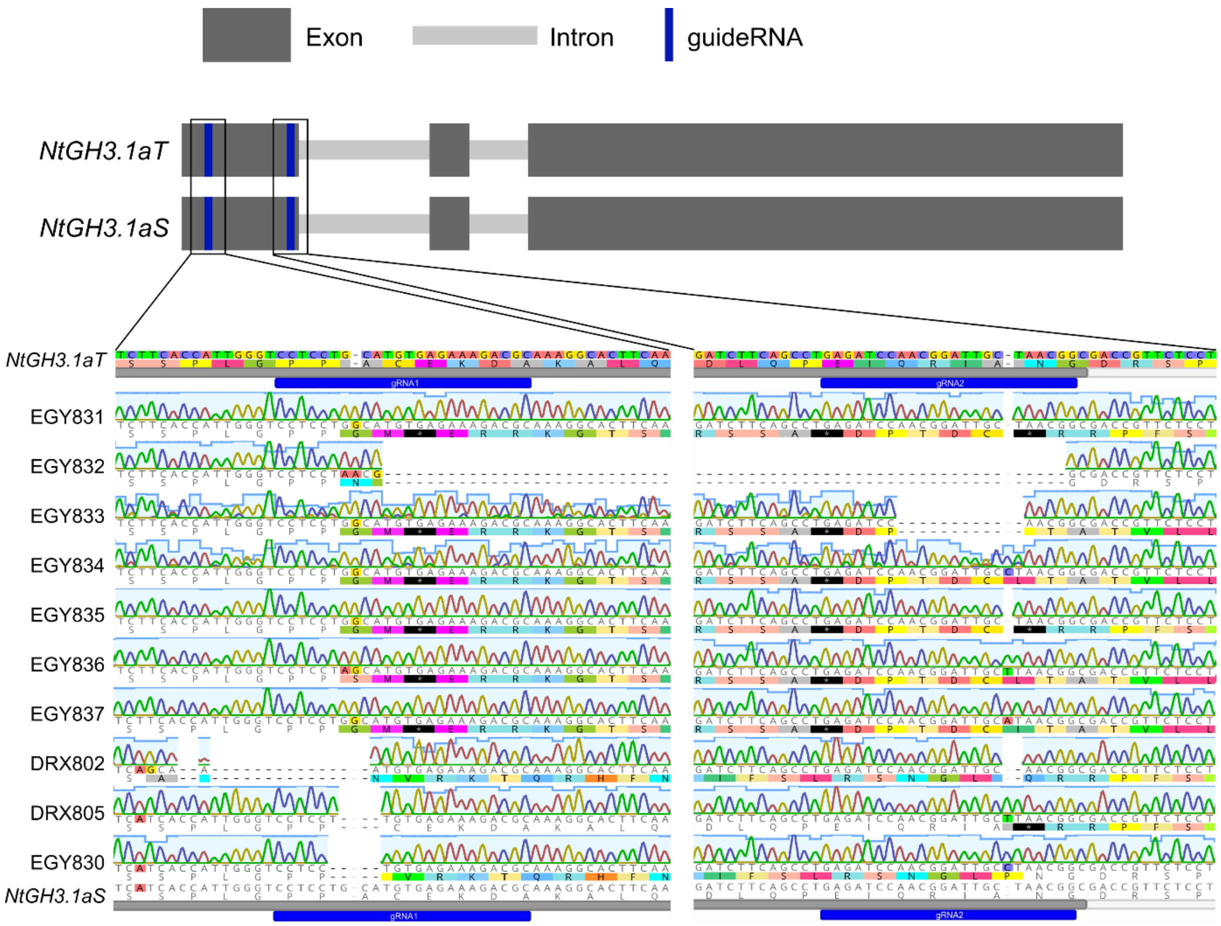

Supplementary Figure S2. Scheme of *NtGH3.1aT/S* genes with targeted guideRNAs and corresponding Sanger sequencing validation. Identification names are listed in Supplemental Table S2 and matching sequences are available on Zenodo.

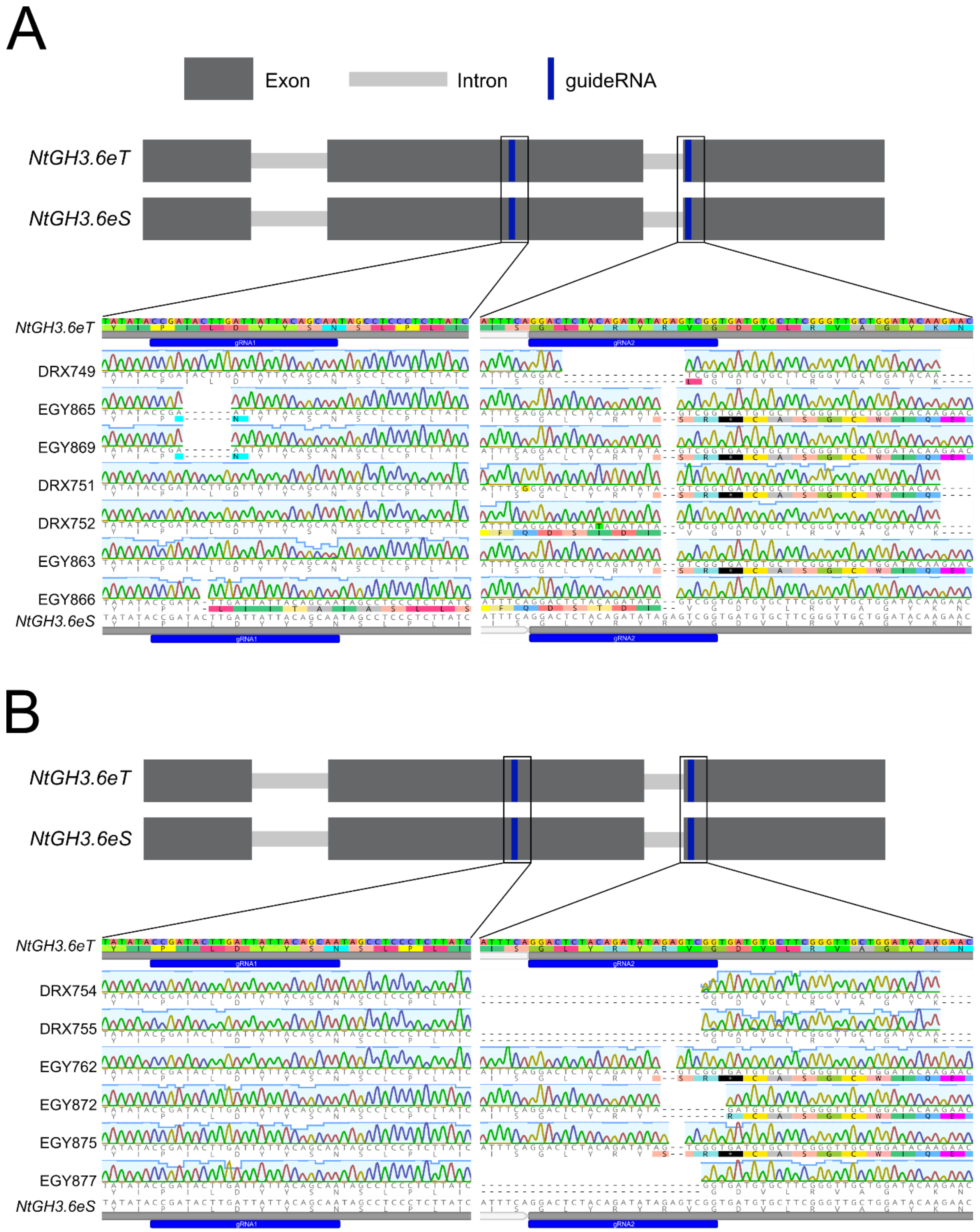

Supplementary Figure S3. Scheme of *NtGH3.6eT/S* genes with targeted guideRNAs and corresponding Sanger sequencing validation. Independent lines of crNtGH3.6e (A) line l1 (B) line l2 are shown. Identification names are listed in Supplemental Table S2 and matching sequences are available on Zenodo.

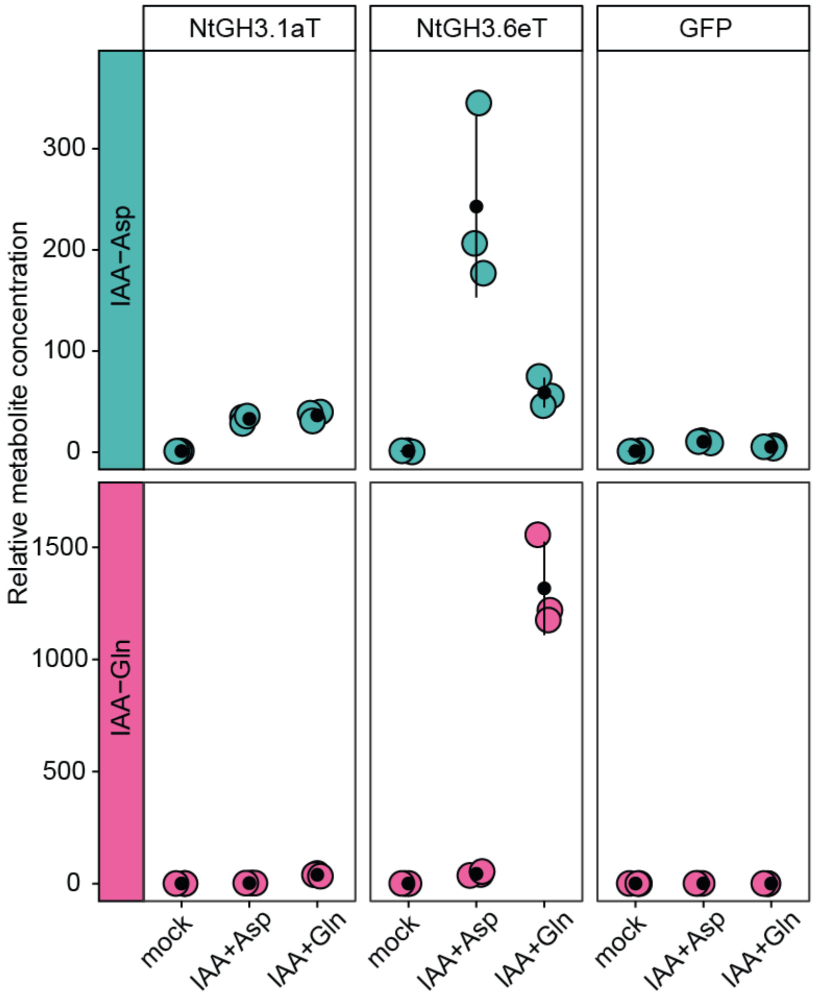

Supplementary Figure S4. Bacterial assay of recombinant tobacco GH3 proteins. Levels of IAA-Asp and IAA-Gln were measured in extracts from NtGH3.1aT- and NtGH3.6eT-expressing bacteria *E. coli* supplemented with IAA and Asp or Gln. Values are related to levels in GFP-expressing bacterial culture, which was used as a negative control. Three biological replications were performed. Data points and error bars indicating mean ± SE are shown.

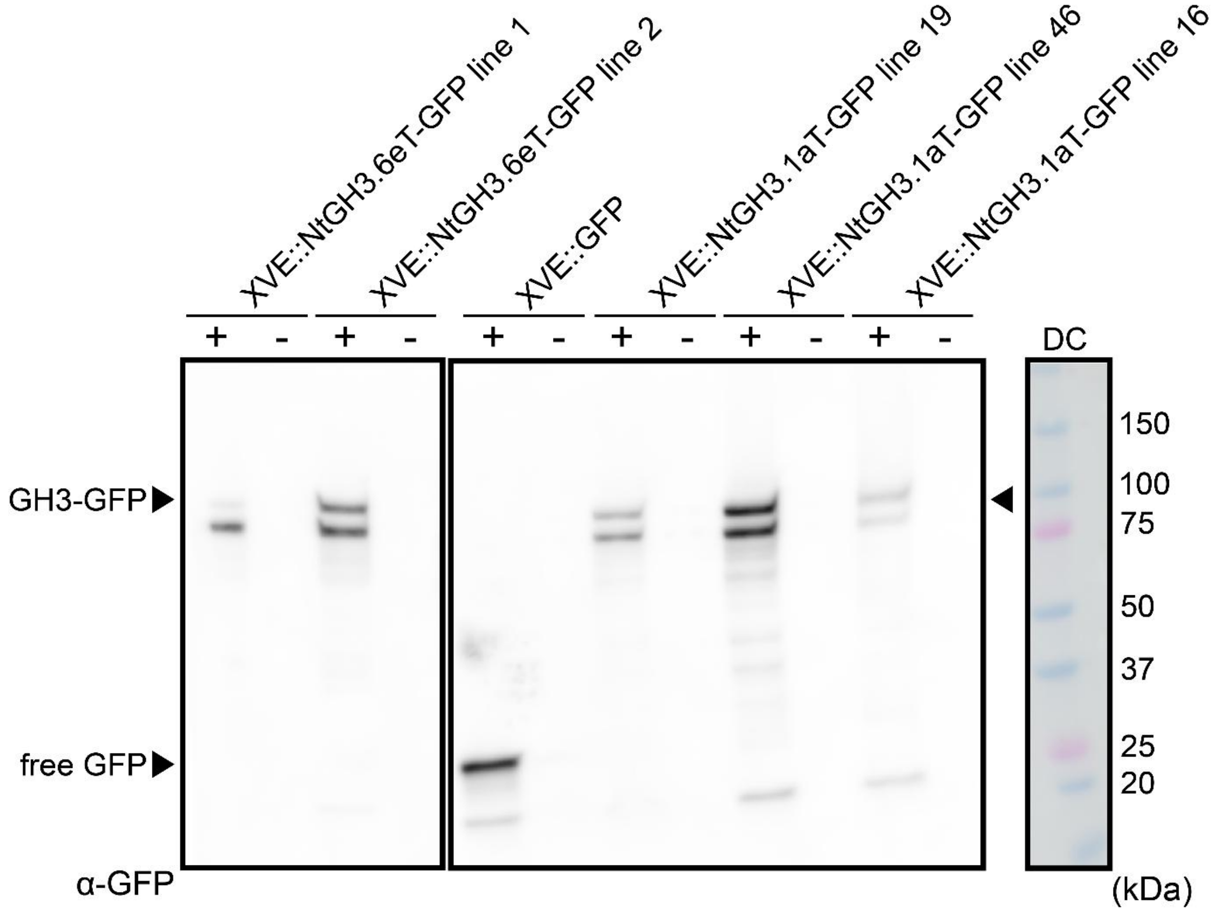

Supplementary Figure S5. Validation of GH3-GFP protein integrity. Western blot analysis was performed on samples from cells overexpressing GH3-GFP fusion or single GFP as a control (XVE::NtGH3.1aT-GFP, XVE::NtGH3.6eT-GFP, XVE::GFP) using anti-GFP antibody. Every sample corresponds to a single line. The symbols (+) and (-) indicate treatment with β-estradiol (induced) or DMSO (mock control), respectively. The predicted size for GH3 protein is approximately 68 kDa while GH3-GFP fusion is approximately 100 kDa.

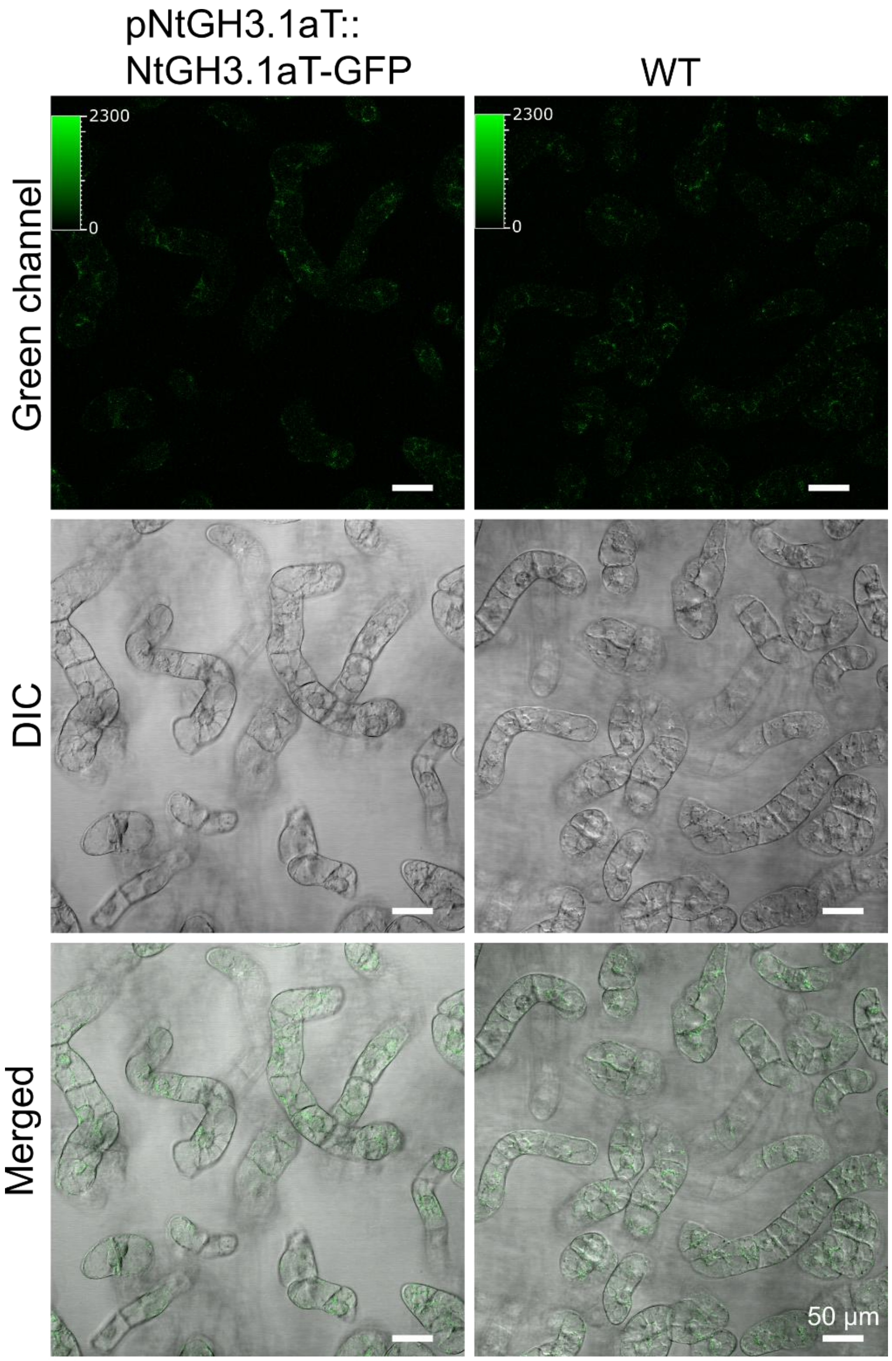

Supplementary Figure S6. Autofluorescence of non-functional line pNtGH3.1aT::NtGH3.1aT-GFP and WT cells. Images are displayed in green channel, differential interference contrast (DIC) and merge of both channels, with lookup tables (LUT) consistent for both lines. Scale bars for all images = 50 µm.

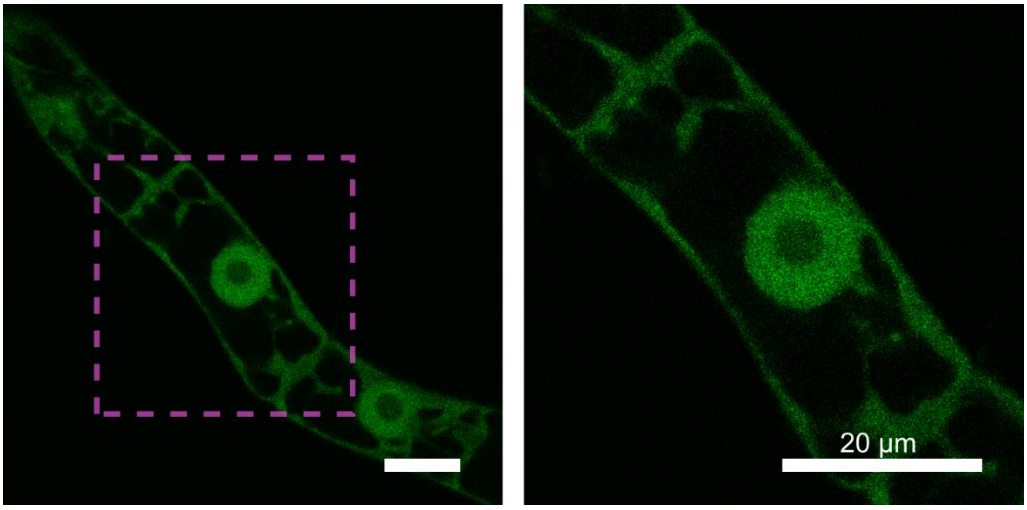

Supplementary Figure S7. Validation of tobacco *NtGH3.1aT* natural promoter functionality**.** GFP driven by *NtGH3.1aT* promoter and terminator was expressed in promoter line pNtGH3.1aT::GFP. Right image is a detail of left image. Scale bars for both images = 20 µm.
